# Data Policy Finder: an easily integratable tool connecting data librarians with researchers to navigate publication requirements

**DOI:** 10.5195/jmla.2024.1865

**Published:** 2024-04-01

**Authors:** Anthony J. Dellureficio, Eric Willoughby, Donna S. Gibson

**Affiliations:** 1 dellurea@mskcc.org, Memorial Sloan Kettering Cancer Center Library, New York, NY; 2 willouge@mskcc.org, Memorial Sloan Kettering Cancer Center Library, New York, NY; 3 donnas9gibson@gmail.com, Memorial Sloan Kettering Cancer Center Library, New York, NY

**Keywords:** Research Data Management, Editorial Policies

## Abstract

The Data Policy Finder is a searchable database containing librarian-curated information, links, direct quotes from relevant policy sections, and notes to help the researcher search, verify, and plan for their publication data requirements. The Memorial Sloan Kettering Cancer Center Library launched this new resource to help researchers navigate the ever-growing, and widely varying body of publisher policies regarding data, code, and other supplemental materials. The project team designed this resource to encourage growth and collaboration with other librarians and information professionals facing similar challenges supporting their research communities. This resource creates another access point for researchers to connect with data management services and, as an open-source tool, it can be integrated into the workflows and support services of other libraries.

## INTRODUCTION

The Data Policy Finder (DPF) is a searchable database containing information, links, direct quotes from relevant policy sections, and notes to help the researcher search, verify, and plan for their publication data requirements. The entries are librarian-reviewed and curated with the intention of adding new content and updating existing information.

Based on conversations with Memorial Sloan Kettering (MSK) researchers about their publishing challenges, a new resource was launched to help them navigate the ever-growing body of policies by publishers regarding data, code, and other supplemental materials. The project team designed this resource to encourage growth and collaboration with other librarians and information professionals facing similar challenges supporting their research communities.

## BACKGROUND

The publication process can often include untimely roadblocks, such as publisher-specific requirements for depositing minimal datasets and identification of datasets, code, and other supporting research materials. These publication policies can be difficult to find, understand, and compare in advance of publishing, and often prolong the publication timeline.

The following use cases have driven the design and resource purpose:

- Researchers are being asked to identify data repositories and expected data output from intended research at increasingly early stages in the research lifecycle. With the National Institutes of Health's (NIH) new Data Management and Sharing Policy, this information would be included in a data management plan. Since the research proposal often presumes a goal of publication, understanding the requirements of intended journals can help researchers align their data plans to lower the eventual burden of publishing.
- Journal publication policies, especially regarding data and other supplemental research output, can be challenging to find and are often buried deep within the interior pages of a publisher's website. They can be presented as PDFs, static HTML pages, or bundled into other author policies. Prospective authors would benefit from a resource to help them discover relevant policies for their specific journal and point them directly to the policies on a publisher's site.

Resource benefits:

- Saves time in identifying publication policy information.
- Highlights critical components of the publisher's policy.
- Links to the complete policy, often difficult to discover.
- Provides curated list of journals in which MSK authors most frequently publish.
- Adds insights and recommendations for repositories via Librarian “Notes.”

**Figure 1:**
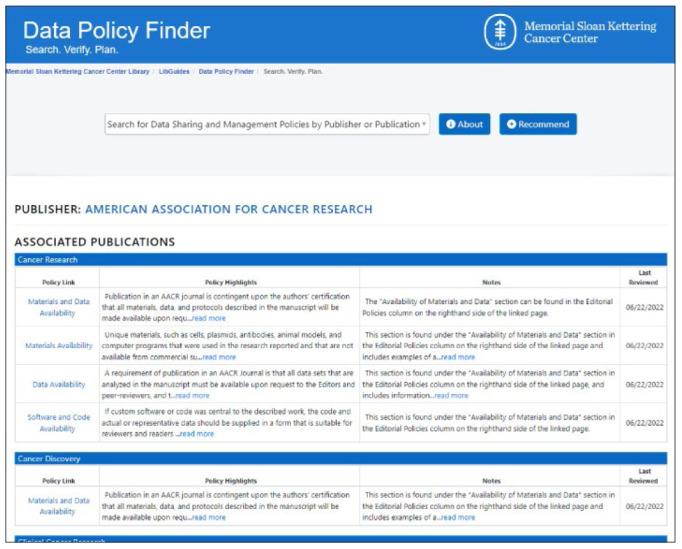
Data Policy Finder

## Searching the Data Policy Finder

Anyone can search and use this resource by simply typing in the name of a Journal or Publisher. Search suggestions based on the entered text will appear and at any point, the user can browse through the list of Publishers and Publications. When selecting a title, the results will display the publisher and all their associated publications with their nested policies.

## Development Process and Decisions

The project team made technology design choices to maximize the versatility of usage, interaction, and integration to facilitate adoption and collaboration by other institutions. The DPF is presented as a basic search, however the user interface is also available in a simplified form connected to a backend application programming interface (API). The minimalist search interface was designed to be embedded in another page while still allowing the user to browse or search existing policies. Separating the backend (data) from the front-end (search interface) allows the DPF to integrate flexibly with an institution's resources and needs, maximizing future growth and integration potential.

Instructions for implementing the DPF are included within the “About” section of the resource.

**Table 1:** Data Policy Finder Record Fields

Policy Link	Type of policy and a link to it on the journal's website
Policy Highlights	Relevant quoted text from the policy
Notes containing	Additional guidance added and curated by Library staff
Last Reviewed	Last date the policy was reviewed or updated in the Data Policy Finder

**Figure 2:**
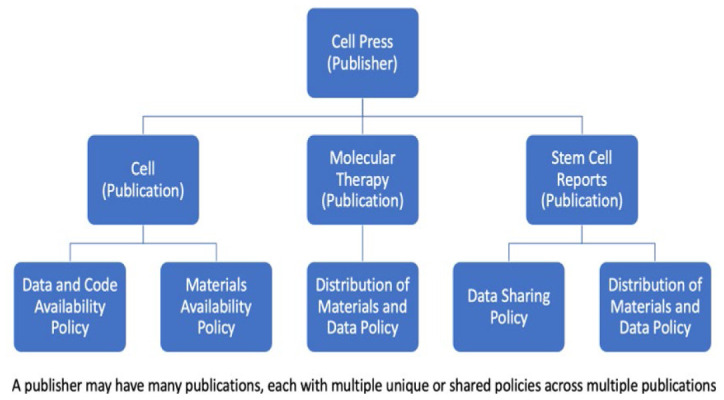
Nested Policy Relationships

## Community Development

The authors believe this resource will bring value to others outside the MSK Library community and are seeking external library partners to collaborate with to enhance resource interface and support timely content revisions and additions. To date, Hofstra/Northwell and Stanford University have incorporated this application within their own resource offerings. The vision of the project team also includes partnerships with publishers and standards organizations.

Since the launch of the Data Policy Finder, the project team are now focus on identifying future enhancements to include:

- Augment the back-end interface
- Capture and track usage metrics
- Add new content
- Create and foster a community for data policies and related initiatives
- Establish metadata standardization
- Develop ingest process for policies—the dream!

Publication policies will continue to grow and change, and researchers can save time using the DPF to better understand manuscript submission requirements for data and associated supplemental materials. This resource creates another access point for researchers to connect with data management services, and as an open-source tool, can be integrated into the workflows and support services of other libraries creating potential collaborations.

## AUTHOR CONTRIBUTIONS

Anthony Dellureficio: conceptualization, data curation, writing – original draft; Eric Willoughby: software, writing – review and editing; Donna Gibson: writing – original draft, writing – review and editing.
